# The effects of vitamin D supplementation on maternal and neonatal outcome: A randomized clinical trial

**Published:** 2015-11

**Authors:** Mahdieh Mojibian, Sedigheh Soheilykhah, Mohammad Ali Fallah Zadeh, Maryam Jannati Moghadam

**Affiliations:** 1 *Department of Obstetrics and Gynecology, Shahid Sadoughi Hospital, Shahid Sadoughi University of Medical Sciences, Yazd, Iran.*; 2 *Department of Endocrine, Shahid Sadoughi University of Medical Sciences, Yazd, Iran.*; 3 *Shahid Sadoughi University of Medical Sciences, Yazd, Iran.*; 4 *Department of Obstetrics and Gynecology, Mojibian Hospital, Yazd, Iran.*

**Keywords:** *Vitamin D*, *Pregnancyoutcome*, *Gestational diabetes*

## Abstract

**Background:**

Vitamin D supplementation during pregnancy has been supposed to defend against adverse gestational outcomes.

**Objective:**

This randomized clinical trial study was conducted to assess the effects of 50,000 IU of vitamin D every two weeks supplementation on the incidence of gestational diabetes (GDM), gestational hypertension, preeclampsia and preterm labor, vitamin D status at term and neonatal outcomes contrasted with pregnant women that received 400 IU vitamin D daily.

**Materials and Methods:**

500 women with gestational age 12-16 weeks and serum 25 hydroxy vitamin D (25 (OH) D ) less than 30 ng/ml randomly categorized in two groups. Group A received 400 IU vitamin D daily and group B 50,000 IU vitamin D every 2 weeks orally until delivery. Maternal and Neonatal outcomes were assessed in two groups.

**Results:**

The incidence of GDM in group B was significantly lower than group A (6.7% versus 13.4%) and odds ratio (95% Confidence interval) was 0.46 (0.24-0.87) (P=0.01). The mean ± SD level of 25 (OH) D at the time of delivery in mothers in group B was significantly higher than A (37.9 ± 19.8 versus 27.2 ± 18.8 ng/ml, respectively) (P=0.001). There were no differences in the incidence of preeclampsia, gestational hypertension, preterm labor, and low birth weight between two groups. The mean level of 25 (OH) D in cord blood of group B was significantly higher than group A (37.9 ± 18 versus 29.7 ± 19ng/ml, respectively). Anthropometric measures between neonates were not significantly different.

**Conclusion:**

Our study showed 50,000 IU vitamin D every 2 weeks decreased the incidence of GDM.

## Introduction

Vitamin D deficiency or insufficiency is common during pregnancy. Maternal vitamin D deficiency in early pregnancy has been linked with a greater risk of gestational diabetes mellitus (GDM), preeclampsia, infections, caesarean section and fetal growth restriction (1). Vitamin D supplementation during pregnancy has been supposed to defend against adverse gestational outcomes (2).

The appropriate dose of vitamin D supplementation during pregnancy is unknown, but it should be more than recommended daily allowance of 200–400IU daily. In 2010, the Food and Nutrition Board at the Institute of Medicine of the National Academies determined that an adequate intake of vitamin D during pregnancy was 600IU per day (3). Hollis *et al* (4), Vieth *et al* (5) and Heaney *et al* (6) have shown that more than 1,000 IU of vitamin D is necessary in pregnancy to sustain a normal circulating range of 25(OH) D. The endocrine society proposed a daily vitamin D intake of 1500–2000IU and a target level of 25 (OH) D of over 30ng/ml (7). However, Hollis and Wagner displayed that by supplementation of pregnant women with 4,000IU daily, 83.9% of women attained a minimal circulating 25 (OH) D level of at least 32 ng/ml (80nmol/ l) at the time of delivery and suggested 4,000 IU of vitamin D is a suitable dose to sustain a maternal serum level of vitamin D over 30 ng/ml and optimize cord blood 25 (OH) D levels (8). Cross sectional studies demonstrated an association between vitamin D deficiency and gestational diabetes (9, 10). Pregnant women with diabetes are known to be more likely to exhibit vitamin D deficiency compared to pregnant women without diabetes (11, 12). Data about the results of vitamin D supplementation on the mother and neonatal outcomes are scant. Some observational studies assessed the association between vitamin D status and pregnancy and neonatal outcomes, but interventional studies about vitamin D supplementation and the effects on Glucose Metabolism, gestational diabetes, preeclampsia; low birth weight and preterm labor are scant.

This study was conducted to assess the effects of 50,000 of Vitamin D supplementation every 2 weeks orally on the primary maternal outcome of GDM, and obstetric outcomes such as gestational hypertension, preeclampsia and preterm labor, vitamin D status at term and neonatal outcomes contrasted with pregnant women that received 400 IU vitamin D daily.

## Materials and methods

A randomized clinical trial was performed on 500 pregnant women with gestational age 12-16 weeks who had serum 25 (OH) D less than 30 ng/ml. These subjects were recruited from two prenatal clinics (Shahid Sadoughi and Mojibian hospitals) in Yazd, Iran between 2010-2012.

Pregnant women with a history of diabetes or subjects who consumed vitamin D supplements during the previous 6 months and women with thyroid or parathyroid disorders were excluded from the study. To detect at least a 3mg/dl difference in the mean of fasting blood glucose at the oral glucose tolerance test ( OGTT) between groups with 80% power, assuming a standard deviation of 10.3, as found in previous our study (13), and _α= 0.05, requires 200 participants per group. We, therefore, aimed to recruit 250 subjects in each group to allow for 20% dropout over the study. All subjects gave written informed consent for participation in the study, which was approved by Ethics Committee of Shahid Sadoughi University of Medical Sciences, Yazd, Iran (reference number=39312). Treatment allocation was made after measurement of baseline plasma 25(OH) D and serum calcium. From the subjects’ medical records, we obtained general information, including maternal age, height, pre pregnancy weight, level of education reproductive and medical histories, and pre pregnancy body mass index (Kg/m^2^). These data were included in the analysis of the data as covariates. The subjects with 25 (OH) D less than 30ng/ml were then divided into two groups randomly. Computer-generated random number lists were drawn up by an independent researcher. Pregnant women and researchers were not blind to treatment assignment. Group A received 400 IU vitamin D (Cholecalciferol) daily, and group B received 50,000 IU every 2 weeks orally. Vitamin D was provided by Zahravi Company, Tabriz, Iran. Safety of 50,000 IU vitamin D during pregnancy was approved by previous our study that demonstrated supplementation with 50,000 IU vitamin D every 2 weeks during pregnancy brought about no adverse effects, such as hypercalcemia or hypervitaminosis in mothers and neonates (13). Supplementation was started in the 12^th^ week of pregnancy and continued until delivery. Pregnant women were followed up every month during pregnancy and were evaluated regarding adverse effects of vitamin D, such as headache and vomiting.

Assessment of primary outcome, such as gestational diabetes was done by 100gr oral glucose tolerance test between 24-28 weeks of pregnancy. The OGTT was performed after an overnight fast of 8 to 14 h while the subject was on an unrestricted diet and unlimited physical activity for at least 3 days. Women were diagnosed with GDM if at least 2 of 4 diagnostic criteria were met (fasting plasma glucose≥ 95 mg/dL, 1-, 2-, and 3-hour plasma glucose levels of≥ 180 mg/dL, ≥ 155 mg/dL, ≥ 140 mg/dL, respectively) (14). Secondary outcomes included maternal obstetric outcomes (gestational hypertension, preeclampsia and preterm labor). Gestational hypertension is usually defined as having a blood pressure higher than 140/90 measured on two separate times, more than 6 hours apart, without the presence of protein in the urine and identified after 20 weeks of pregnancy Preeclampsia, eclampsia was defined as hypertension develops after the 20th week of pregnancy and is accompanied by proteinuria. Preterm labor was diagnosed as regular uterine contractions occurring before 37 weeks gestation that are accompanied by a progressive cervical change. We assessed other outcomes such as, serum level of 25 (OH) D at the time of delivery from mother and cord, neonatal weight, length, head circumference and Apgar of 1 and 5 minute. Other neonatal complications such as macrosomia, respiratory distress and hypoglycemia also were assessed.

Plasma glucose was measured using an enzymatic in vitro test (GOD-PAP kit, Pars Azmun Co. Inc, Tehran, Iran) and 25 (OH) D was analyzed by Eliza (Euroimmun Kit, Nima Pooyesh Teb Company, Tehran, Iran) with an inter-assay coefficient of variation of 7.8% and an intra assay coefficient of variation of 3.2%.

**Statistical analysis**

Statistical analysis was performed using the Statistical Package for the Social Sciences, version 17.0, SPSS Inc, Chicago, Illinois, USA (SPSS software). For continuous data, Student´s *t-*test was used for normally distributed data and a nonparametric test (Mann-Whitney) for non- normally distributed data. The Chi-square test was used for categorical variables. A p-value of <0.05 was considered statistically significant.

## Results

**Figure 1:**
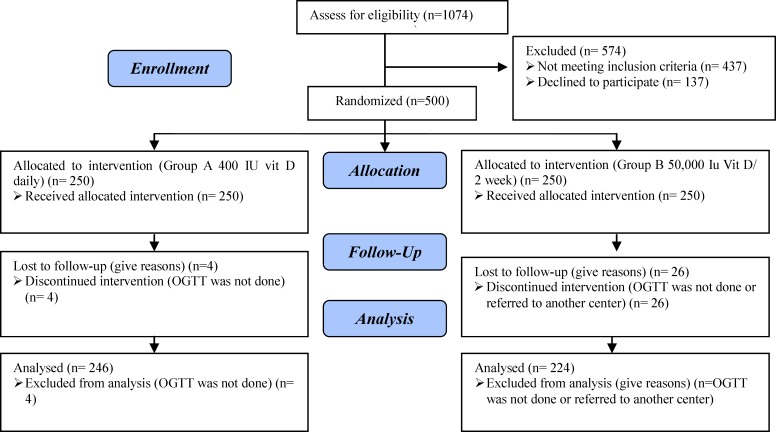
Flow chart of the subjects throughout the study for primary outcome.

**Figure 2:**
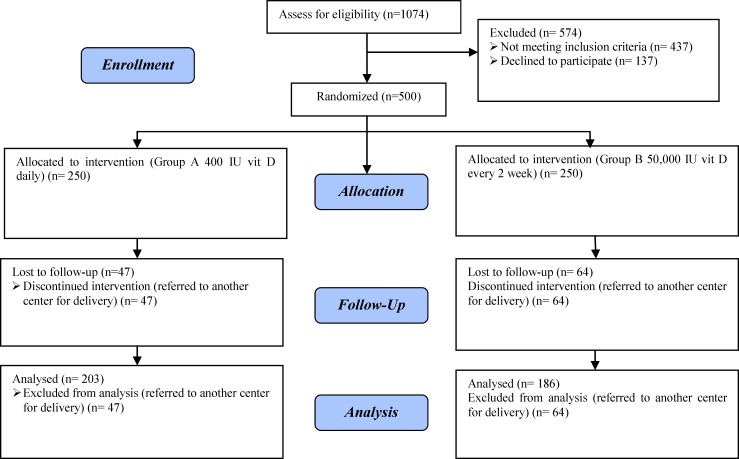
Flow chart of the subjects throughout the study for secondary outcome

1074 mothers consented to participate and from these pregnant women 500 were randomly assigned to treatment groups. After allocation, 30 subjects (6%) discontinued intervention (OGTT was not done or referred to another center) and 470 were followed up to assess primary outcome (Figure 1). From 500 pregnant subjects 389 completed participation for assessment of secondary outcomes and 111 pregnant women discontinued intervention because they referred to another center for delivery (Figure 2). 

Table I illustrates the characteristics of the two groups. In this study pre-pregnancy BMI, age, number of pregnancies, previous history of GDM, family history of diabetes, level of education, Seasons of blood sampling did not show a significant difference between two groups.

None of the subjects smoked cigarettes. The serum levels of 25 (OH) D and serum calcium before intervention between the two groups were not significantly different (Table I). The mean level of vitamin D was 14.9± 6.3 ng/ml.

**Primary outcome**

In this study, 246 subjects from group A and 224 pregnant women from group B completed participation for assessment of primary outcome. The mean±SD level of fasting plasma glucose, 1-h, 2-h and 3-h blood glucose at OGTT were significantly lower in group B than group A (Table II). This study demonstrated the incidence of GDM in our participants was 10.4%. The occurrence of GDM in the group that received 50,000 IU vitamin D every 2 weeks was significantly lower than pregnant women who were supplemented with 400 IU of vitamin D daily, 6.7% versus 13.4% and Odds ratio (95% Confidence interval) of GDM in group B compared to group A was 0.46 (0.24-0.87) (p=0.01) (Table II). The sample size of our study was enough because it had 80% power to detect a 50% decrease in risk of GDM.

**Secondary outcomes**

In this study, 203 subjects from group A and 186 pregnant women from group B completed participation for assessment of secondary outcomes.

The mean level of 25 (OH) D at the time of delivery in group B was significantly higher than group A (37.9± 19.8 versus 27.2± 18.8) (P=0.001). 

In group B, serum 25(OH) D levels above 30ng/ml at the time of delivery and in cord blood was found in 51.9% and 56.3%, respectively. In group A, it was considered 29.5 and 26.4%, respectively (p=0.001). No significant difference was demonstrated between two groups in term of gestational hypertension, preeclampsia and premature labor and low birth weight (Table II).

The mean level of vitamin D in cord blood in group B was higher than group A (37.9±18 versus 29.7 ± 19, respectively) (p=0.005). No maternal or cord blood hypervitaminosis (25 OH) D more than 100ng/ml was demonstrated. The results indicated the mean level of neonatal weight, length, head circumference and Apgar in minutes1 and 5 between two groups were not significant different (Table II). In this study two neonates (1.2%) developed hypoglycemia in group B and eight neonates (4.1%) in group A (p= 0.07). Respiratory distress was observed in four neonates that needed a ventilator (2 neonates in each group (p= 0.6).

**Table I:** Baseline characteristics of pregnant women in two groups

**P-value**	**Group B (50,000IU Vit D every 2 weeks)** **n=250**	**Group A (400IU Vit D/daily)** **n=250**	**Variables**
0.4	27.8±5	27.3±4.9	Age (year)*
0.06	25.9±4.8	26.8±4	Before pregnancy BMI (Kg/m2)*
0.13	1.8±0.8	2.1±1.1	Number of pregnancies (n)*
0.26	0.8%	2.7%	Previous history of GDM (%)
0.14	50.8%	58.3%	Family history of diabetes (%)
0.22	9.3% 90.7%	5.6% 94.4%	Education Diploma or less (%) Higher than diploma (%)
0.64	16.7 25.5 28.4 29.4	15.4 31.8 23.4 29.4	**Season of blood delivery (%)** Spring Summer Autumn Winter
0.14 0.9	14.46±5.19 8.9±0.4	15.31±5.19 9±0.5	25 (OH) D (ng/ml) * Serum calcium ( mg/dl)*

Chi-Square test and Student’s *t*- test were used to compare variables between two groups.

BMI: Body Mass Index; GDM: Gestational diabetes mellitus; 25 (OH) D: 25-hydroxyvitamin D.

* Data were presented as Mean ±SD

**Table II:** Primary and secondary outcomes in two groups

**Variable**	**Group A (400 IU Vit D)**	**Group B (50,000 IU Vit D every 2 weeks)**	**p-value**
25 (OH) D3 of pregnant women at delivery time (ng/ml) (Mean±SD)	27.2±18.8	37.9±19.8	0.001
**OGTT Results at 24-28 weeks**			
Number Fasting blood glucose (mg/dl) (Mean±SD) 1-h OGTT 2-h OGTT 3-h OGTT	246 86.4±12.9 164.4±35.6 137.3±33.9 108.6±31.2	224 82±11.9 153.4±33.7 127.1±30.6 94.1±24.4	0.004 0.022 0.023 0.001
GDM (%)	13.4	6.7	0.01
**Maternal outcomes**			
Number Gestational hypertension (%) Preeclampsia (%) Preterm delivery (%)	203 0.7 2 4.2	186 1.3 3.9 5.2	0.57 0.33 0.58
**Neonatal outcomes** ( Mean±SD)			
Number Birth length (cm) Birth weight, (g) Head circumference(cm) Apgar (minute1) Apgar (minute 5)	203 50.22±5.4 3125.57±434.9 34.31±2.8 9.7±0.7 9.8±0.5	186 50.39±2.1 3088.72±481.21 34.35±2.2 9.83±0.6 9.9±0.2	0.7 0.43 0.88 017 0.17
25( OH) D3 of cord blood( ng/ml) (Mean±SD)	29.7±19	37.9±18	0.005
**Neonatal complication** (%)			
Low birth weight Macrosomia Hypoglycemia Respiratory distress	4 1.5 4.1 1	5.9 1.6 1.2 1.2	0.25 0.61 0.07 0.67

Chi-Square test and Student’s *t*- test were used to compare variables between two groups.

25 OH D3: 25-hydroxy vitamin D; OGTT: oral glucose tolerance test; GDM: Gestational diabetes mellitus

## Discussion

Low levels of serum 25 (OH) D have been reported in many populations, including pregnant women. Studies have demonstrated associations between low levels of serum 25 (OH) D in pregnancy and maternal/ neonatal health outcomes. However, many of these studies are observational in nature. The aim of the present study was to contrast the maternal and neonatal outcomes in the group supplemented with 400 IU of vitamin D daily (group A) with the subjects that received 50,000 IU of vitamin D (Cholecalciferol) orally every 2 weeks

**Primary outcome**

**GDM**

Low serum 25 (OH) D concentration has been associated with an impaired glucose tolerance test and gestational diabetes (11, 12, 15-17). Interventional studies about the effect of vitamin D supplementation on GDM are scare. The results of observational studies revealed association of vitamin D deficiency and GDM. Poel *et al* in a systematic review and meta-analysis showed that maternal vitamin D status is was correlated with gestational diabetes (18). Aghajafari *et al* in a systematic review and meta-analysis of observational studies showed that vitamin D insufficiency is was associated with greater risk of gestational diabetes, preeclampsia, and small for gestational age infants. This study also indicated that pregnant women with low serum vitamin D had a heightened risk of lower birth weight infants (19). In our randomized clinical study, it was shown that the incidence of gestational diabetes in pregnant women who were supplemented with 50,000 IU vitamin D every 2 weeks was lower than the group that received 400 IU vitamin D daily. But Yap *et al* showed that supplementation with 5000 IU vitamin D3 daily at a mean 14 weeks gestation did not cause to improvement in maternal glucose level compared with a control group (400 IU of vitamin D3 daily) (20). This difference could be due to the higher number of pregnant women that participated in our study (470 versus 179).Soheilykhah *et al* demonstrated that insulin resistance significantly decreased when pregnant women were supplemented with 50,000 IU of vitamin D every two weeks from 12 weeks of pregnancy until delivery (13). In our study, the mean level of fasting plasma glucose, 1-h, 2-h and 3-h blood glucose at OGTT were significantly lower in group B than group A, but the results of the study conducted by Soheilykhah *et al* showed FBS was not significantly different after supplementation with 50,000 IU of vitamin D3 (13). This difference may be due to the number of subjects in our study. 

In the study performed by Hossain *et al* it revealed that pregnancy outcomes such as gestational diabetes, gestational hypertension, and preterm in pregnant women that were supplemented with 4,000 IU of vitamin D from 20 weeks until delivery showed no significant difference compared to the control group (21). Asemi *et al* in a randomized clinical trial on 54 pregnancies with gestational diabetes showed 50,000 IU vitamin D 2 times during the study (at baseline and at day 21 of the intervention) led to a significant decrease in concentrations of fasting plasma glucose and serum insulin compared to the control group (22). Different mechanisms have been advanced for the decrease of insulin resistance in response to the improvement of vitamin D status. Vitamin D increases cellular glucose absorption either directly or by increasing insulin sensitivity (23).

**Secondary outcomes**

**Level of vitamin D at the** **time of delivery**

In our study the level of vitamin D at the time of delivery in mothers were significantly increased. Regil *et al* in a systematic review and meta analysis of thirteen RCTs (n =2,299) confirmed that serum 25 (OH) D levels in supplemented group were significantly higher at term, compared with the control group (mean difference: 66.5 nmol/L, 95% CI 66.2–66.7) (2). In our study, supplementation with 50,000 IU vitamin D was able to raise serum 25 (OH) D 30 ng/ml in mothers at the time of delivery and prevented neonatal vitamin D deficiency. Our results were similar to the study conducted by Yap *et al* that indicated mean neonatal cord 25 (OH) D was higher in neonates of the group that supplemented with 5000 IU D3 compared to 400 IU D3 daily (20). In our study in the supplemented group, serum (OH) D levels above 30ng/ml at the time of delivery and in cord blood was found in 51.9% and 56.3%, respectively, but Yap showed 10% of high dose women remained vitamin D deficient. This difference could be related to the lower baseline level of 25(OH) D in our subjects.

**Birth weight, length, head circumference, One-and 5-minute Apgar scores.**

Vitamin D is known to impact the obtainment of bone mineral in utero, and changes in women’s calcium homeostasis during pregnancy promote calcium supply for bone mineralization in the rapidly growing fetal skeleton (24). Mother-offspring cohort studies have shown that maternal vitamin D insufficiency has a negative effect on bone mineral accrual by the fetus, leading to decreased bone mass at birth and in childhood (25). Mahon *et al *showed that the vitamin D status of mothers during pregnancy is correlated with the morphology of the developing fetal femur (26). 

In our study, birth weight and length were not significantly different in two groups and our results which were similar to Brook *et al* study that found no significant difference in offspring birth length in UK Asian women that supplemented with 1000 IU ergocalciferol daily at third trimester compared to the control group (27). Hollis *et al* randomized 350 pregnant women to 400 IU/day, 2,000 IU/day or 4,000 IU/day of oral vitamin D3 from 12-16 weeks of pregnancy until delivery. Maternal serum vitamin D at delivery was higher in women receiving the higher dose of vitamin D but there was no significant difference in neonate birth weight between the three groups (8). In contrast, Marya *et al* showed that birth length was significantly higher in women supplemented with a much higher dose of vitamin D (two doses of 600,000 IU cholecalciferol in the 7th and 8th month of gestation), compared to the unsupplemented women (28). Delvin *et al* and Mallet *et al* studies also did not demonstrate a significant difference in offspring birth weight with maternal vitamin D supplementation (29, 30). In our study, head circumference of neonates were not significant difference between two groups that supplemented with 400 IU vitamin D daily or 50,000 IU vitamin D every two weeks and our results were similar to Brook *et al* study (27), but Marya *et al* randomized 200 Indian women to either no supplement or to two doses of 600,000 IU cholecalciferol in the last trimester and found that head circumference at birth was significantly higher in the supplemented group compared to the unsupplemented group (28). Our results indicated that the mean level of neonatal weight, length and head circumference between the two groups were not significantly different and our results was in concordance with Yap *et al* that showed the anthropometric measures birth weight, crown-heel length and head circumference were not different between two supplemented groups (400 IU vitamin D versus 5000 IU vitamin D daily) (20). Maghbooli *et al* demonstrated that there was not a significant correlation between maternal and cord blood vitamin D concentration and newborn weight, height, head circumference and Apgar scores (31). Saboor *et al*, however, confirmed that there was a significant correlation between sufficient maternal calcium and vitamin D intake, length, birth weight, the 1 min Apgar score and weight gain of the mother (32).

In our study, 1, 5 minute Apgar scores showed no significant differences between two groups, but Hossain *et al* demonstrated one -and 5-minute Apgar scores were significantly higher in the supplemented group compared to control but neonatal anthropometric parameters were not different between the two groups (21). The difference of results may be due to heterogeneity of method of studies, population, time and dose of supplementation during pregnancy. 

**Preeclampsia, Preterm labor and low birth weight**

The most data relating maternal 25 (OH) D level and risk of offspring preterm birth, preeclampsia and low birth weight are observational. Similar to many other outcome measures, results of the different observational studies were conflicting, with some showing an inverse correlation between maternal vitamin D level and risk of preeclampsia and others no relationship. However, there was significant heterogeneity between studies about gestational age that maternal vitamin D status was evaluated, confounding factors adjusted for and the definition of preeclampsia used. Most observational studies were case-control and included small numbers of cases of preeclampsia. Our study demonstrated the incidence of preeclampsia, preterm labor and low birth weight were not influenced by vitamin D supplementation. Marya *et al* in a clinical trial randomized 400 pregnant women to either a trial of vitamin D plus calcium (375mg/day calcium plus 1200 IU vitamin D) from 20-24 weeks until delivery or no supplement (n=200 in each arm). No difference in the risk of preeclampsia was identified in the un-supplemented group (28 ), but Sablock *et al* showed forty-four percent patients in unsupplemented group and 20·3% patients in the supplemented group developed preterm labor/preeclampsia/gestational diabetes. 

Newborns of mothers in un-supplemented group had lower cord blood levels of 25 (OH) D levels and lower birth weight as compared to control group (33). 

Ferandez-Alfonso *et al* in a Spanish cohort study showed that there was no significant difference in mean maternal 25 (OH) D concentration measured at 11-14 weeks in women who delivered preterm and those who delivered at term (34).

A sufficient vitamin D status may also protect against other adverse pregnancy outcomes. For pregnant women who were supplemented with vitamin D, data from the three trials studies revealed a trend for lower possibility of birth weight below 2500 gr (23, 27, 35). 

Some observational studies indicate that vitamin D levels during pregnancy can have an effect on the fetal bone development and growth of children (36-38). Burris *et al* showed that second trimester 25 (OH) D levels under 25nmol/l were associated with a higher occurrence of small for gestational age (39). 

**Safety**

Our study indicated that supplementation of pregnant women with 50,000IU vitamin D every 2 weeks caused no adverse consequences for pregnant women and the level of vitamin D reached over 30ng/ml, it remained below the toxic level (more than 100 ng/ml) at the time of delivery and these results are in concordance with the Hollis *et al* study that presented 4000 IU daily is safe during pregnancy (8). Yap *et al* showed the safety of high dose supplementation at a dose of 5000 IU daily in women with baseline plasma 25(OH) D level less than 32 ng/ml (18). Soheilykhah *et al* demonstrated that supplementation with 50,000IU vitamin D every 2 weeks brought about no adverse effects, such as Hypercalcemia, in pregnant women (13).

However, our study was a randomized clinical trial in an appropriate population, but it has limitations. The pregnant women and researchers were not blind to treatment, which may have performance bias. We also did not measure the calcium concentration in neonates and could not able to diagnosis asymptomatic hypercalcemia. The number of subjects who developed preeclampsia, preterm labor and low birth weight was low and we need to perform a blind randomized clinical trial in a large population to assess the effect of vitamin D supplementation on these outcomes. 

## Conclusion

Our study showed that consumption of 50,000 IU vitamin D every 2 weeks from 12 weeks of pregnancy until delivery the incidence of GDM significantly reduced, but the incidence of preeclampsia, preterm labor, low birth weight and anthropometric parameters of neonates were not significantly different in the supplemented groups. This study also demonstrated that vitamin D supplementation with 50,000 IU every 2 weeks in pregnant women with baseline level less than 30ng/ml could prevent vitamin D deficiency in neonates. This dose was safe without adverse effects.

## Note

***Registration code: IRCT201107237097N1***
